# Functional Filaments: Creating and Degrading pH-Indicating PLA Filaments for 3D Printing

**DOI:** 10.3390/polym15020436

**Published:** 2023-01-13

**Authors:** Shelbie A. Legett, John R. Stockdale, Xavier Torres, Chris M. Yeager, Adam Pacheco, Andrea Labouriau

**Affiliations:** Los Alamos National Laboratory, Bikini Atoll Road, Los Alamos, NM 87545, USA

**Keywords:** PLA, polylactic acid, pH indicators, additive manufacturing, polymer composites, biodegradation, fused filament fabrication, FFF, PLA aging

## Abstract

With the rapid pace of advancements in additive manufacturing and techniques such as fused filament fabrication (FFF), the feedstocks used in these techniques should advance as well. While available filaments can be used to print highly customizable parts, the creation of the end part is often the only function of a given feedstock. In this study, novel FFF filaments with inherent environmental sensing functionalities were created by melt-blending poly(lactic acid) (PLA), poly(ethylene glycol) (PEG), and pH indicator powders (bromothymol blue, phenolphthalein, and thymol blue). The new PLA-PEG-indicator filaments were universally more crystalline than the PLA-only filaments (33–41% vs. 19% crystallinity), but changes in thermal stability and mechanical characteristics depended upon the indicator used; filaments containing bromothymol blue and thymol blue were more thermally stable, had higher tensile strength, and were less ductile than PLA-only filaments, while filaments containing phenolphthalein were less thermally stable, had lower tensile strength, and were more ductile. When the indicator-filled filaments were exposed to acidic, neutral, and basic solutions, all filaments functioned as effective pH sensors, though the bromothymol blue-containing filament was only successful as a base indicator. The biodegradability of the new filaments was evaluated by characterizing filament samples after aging in soil and soil slurry mixtures; the amount of physical deterioration and changes in filament crystallinity suggested that the bromothymol blue filament degraded faster than PLA-only filaments, while the phenolphthalein and thymol blue filaments saw decreases in degradation rates.

## 1. Introduction

Poly(lactic acid), or PLA, is one of the most popular polymers used for fused filament fabrication (FFF) due to its desirable mechanical characteristics as well as its biodegradability and ability to be synthesized from renewable resources [1]. Currently, most PLA is created by the polymerization of lactic acid monomers derived from starch feedstocks such as corn or sugar rather than petroleum-based feedstocks. With production systems reportedly requiring 25–55% less energy than petroleum-based polymers, PLA is currently one of the most promising “green” plastics on the market [1,2,3].

The degradation of PLA in natural environments such as soils/landfills occurs in two main steps: abiotic hydrolysis and microbial decomposition [3,4,5]. During the abiotic hydrolysis stage, water severs ester linkages within the polymer main chain leading to fragmentation of the polymer and a decrease in molecular weight [6]. This is typically a very slow process under ambient environmental conditions (hundreds to thousands of years) [7], but the exact rate and extent of hydrolysis depends on a variety of inherent and external factors. Inherent factors affecting PLA degradation include the lactic acid isomer ratio, crystallinity, and molecular weight of the PLA, while external influences include environmental temperature, humidity, pH, and UV exposure. Lactic acid exists in nature as two stereoisomers: _L_-lactic acid and _D_-lactic acid. When PLA is produced via fermentation of natural starch feedstocks (most PLA is currently produced using this method) as opposed to synthesis from petrochemicals, the end product is 99.5% _L_-lactic acid and 0.5% _D_-lactic acid, while petrochemical synthesis produces a roughly 50–50 mixture of _L_- and _D_-isomers [3,5,8,9]. PLA consisting of >94% _L_-isomers is semi-crystalline, leading to a higher resistance to degradation by hydrolysis compared to the more amorphous PLA containing greater amounts of _D_-isomers [3,5,10,11,12,13]. Additionally, PLAs with higher molecular weight degrade more slowly than lower molecular weight PLAs due to the greater number of ester linkages that need to be destroyed through hydrolysis [3,5,6]. 

External conditions such as temperature, humidity, pH, and UV exposure also effect the rate and extent of PLA degradation. Elevated temperatures—especially above the glass transition temperature of PLA (55–62 °C)—and higher humidity cause an increased water adsorption and more flexibility in the polymer chains, leading to faster rates of hydrolysis as well as greater microbial attachment [3,5,11,12,14,15,16,17]. As a result of this, industrial composting, which is typically performed at 60–70 °C, can degrade PLA in a matter of weeks or months compared to the hundreds of years needed to reach the same level of degradation under ambient environmental conditions [7,18,19,20,21]. Alkaline conditions lead to faster PLA hydrolysis due to the increased concentration of hydroxide ions [3,13,22]. UV light also increases the rate of PLA degradation by causing brittleness and lowering the molecular weight [3,23,24,25,26,27]. As the molecular weight and crystallinity of PLA decreases due to abiotic processes, the PLA matrix is more amenable to enzymatic attack and microbial degradation begins; microorganisms continue PLA decomposition by ultimately assimilating the lactic acid monomers and releasing CO_2_ [3,6]. 

To impart enhanced mechanical properties or additional functionalities, PLA is often combined with other materials such as thermoplastic polyurethane [28,29], poly(ethylene glycol) [30,31], graphene [32,33,34], and ceramics [35,36,37,38,39]. However, when creating new PLA composites for applications such as additive manufacturing, one must not only consider the effects of the filler on the thermomechanical properties of PLA (i.e., [29]), but also the fillers’ effects on the end-product’s biodegradability. For example, antimicrobial properties can be imparted to PLA by adding ceramic fillers such as titanium- and zinc-oxides [35]. The resulting PLA composite will have a higher degradation temperature and higher crystallinity than unmodified PLA, leading to overall slower degradation rates [35,36,37,40]. This result may be desirable for end products such as medical implants and outdoor furniture that require long-term stability, but it would be a negative characteristic for items destined for single-use consumption such as packaging or eating utensils. 

Recently, the incorporation of pH indicators in PLA-based composites has been investigated in the context of creating packaging capable of signifying changes in food freshness [41,42,43,44]. For this application, both the PLA composite and the indicator must be food-safe, leading to the use of indicators derived from plants [42,44] or microorganisms [43]. Though biodegradation was a consideration in these studies, the effects of the indicators on the degradation of the PLA was not investigated [41,42,43,44]. Additionally, there are currently no commercially available pH-indicating filaments for 3D printing. In this study, PLA was melt-blended with three common pH indicators—bromothymol blue (BB), phenolphthalein (PP), and thymol blue (TB)—as well as poly(ethylene glycol) (PEG) to create functional filaments that can be used to print everyday objects capable of informing users of pH changes in their environments. Along with determining their pH indicating abilities, these new filaments were characterized via physical, chemical, thermal, and mechanical analyses and finally tested for degradability through soil and soil-slurry aging experiments. 

## 2. Materials and Methods

### 2.1. Materials

**Figure 1:**
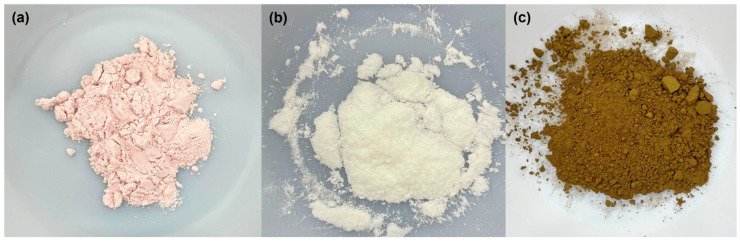
pH indicator powders used in this study: (**a**) bromothymol blue, (**b**) phenolphthalein, and (**c**) thymol blue.

The base of the filaments was made from PLA pellets (Luminy^®^ LX175; Total Corbion PLA, Gorinchem, 4206 AC, The Netherlands). The fillers used include acid/base indicators (bromothymol blue, phenolphthalein, and thymol blue (ACROS Organics, Geel, Belgium)) along with the plasticizer poly(ethylene glycol) (MW = 2000 Da; Sigma-Aldrich Inc., St. Louis, MO, USA) to counter the brittleness caused by adding powders to PLA. The coloration and physical properties of the acid/base indicators can be seen below in Figure 1 and Table 1. These indicators were chosen because they all had melting points above the typical extrusion temperature of PLA, as well as a variety of pH-indicating ranges from very acidic (TB) to neutral (BB and TB) to very basic (PP and TB). Hydrochloric acid and ammonium hydroxide (Thermo Fisher Scientific, Waltham, MA, USA) were used to test the pH-indicating functionality of the filaments. A purge compound (ASACLEAN™ E; Asahi Kasei Corporation, Tokyo, Japan) was used to clean the microcompounder before making each filament. Ultra-high purity nitrogen (Airgas, Radnor, PA, USA) was used for thermogravimetric analysis (TGA) and differential scanning calorimetry (DSC). Deuterated chloroform (99.6% deuterated; ACROS Organics, Geel, Belgium) was used to prepare samples for nuclear magnetic resonance (NMR) spectroscopy.

### 2.2. Filament Fabrication

**Figure 2:**
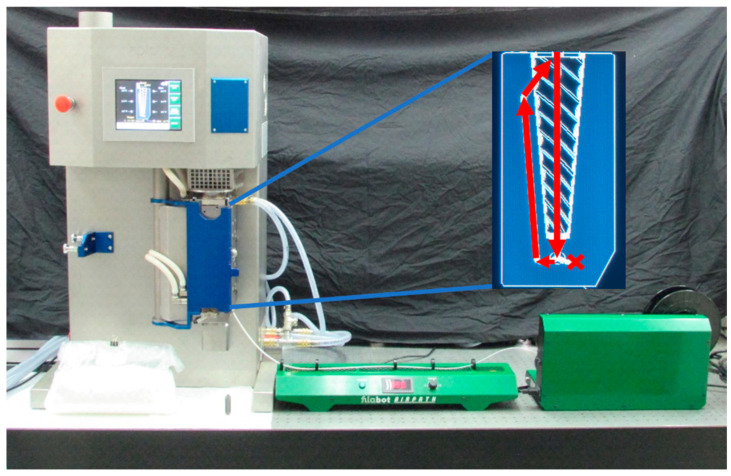
Filament extrusion setup with Xplore MC 40 microcompounder, Filabot Airpath, and Filabot Spooler. Inset: Graphic representation of material pathway through microcompounder in recirculation mode.

There are different methods one can employ to create new filaments for FFF. The homogeneity of the resulting filament is very important to ensure printability and optimal functionality; therefore, researchers have come up with various ways to uniformly distribute fillers within a PLA matrix, including adding fillers to PLA dissolved in solvent—the method employed in our group’s previous work [35]—and repeated extrusion and recycling of PLA filler composites. In this study, filaments were created using a twin-screw microcompounder and extruder (Xplore MC 40; Xplore Instruments BV, Sittard, The Netherlands; Figure 2) allowing the PLA and fillers to melt and mix thoroughly within the machine before being extruded. This not only ensures filament homogeneity, but also eliminates waste generated by solvent mixing and saves a considerable amount of time compared to solvent mixing and composite recycling.

The filaments created in this study are listed in Table 2. To avoid degradation of the PLA feedstock due to humidity, PLA pellets were dried at 45 °C for 4 h and stored in a desiccator, along with the PEG flakes, until ready for use. For all composite filaments, the measured components were sealed in glass jars and shaken to evenly distribute PEG and indicator powders among the PLA pellets. The mixing screws were set to run in parallel (both screws rotating in the same direction) for all filaments. Before creating each new filament, the microcompounder was cleaned by flushing with a purge compound at 180 °C and 100 rpm until the compound extruded was white. Next, the instrument was flushed with raw PLA pellets at 180 °C and 100 rpm until all of the purge compound was extruded and the PLA emerged colorless and translucent. Finally, all “flushing” PLA was removed until the mixing chamber of the microcompounder was empty.

After cleaning, the microcompounder was switched to “recirculation” mode (blocking extrusion and allowing contents to be circulated within the compounder; Figure 2 inset), and the twin-screw rotation speed was lowered to 50 rpm. Half of the filament mixture was then added to the microcompounder and allowed to compound at 180 °C until the torque reported by the machine was stabilized at <1 Nm, indicating the homogenization of the melted components. At this point, the temperature was adjusted to the values listed in Table 3, and the microcompounder was switched to “extrusion” mode, allowing the molten material to flow out of the instrument. To ensure continuous material expulsion, the remaining half of the PLA mixture was added slowly throughout the extrusion. Following extrusion, the filament was cooled by positioning it over fans in a Filabot Airpath (Filabot, Barre, VT, USA), and the solidified filament was spooled using a Filabot Spooler (Figure 2).

The microcompounder was thoroughly cleaned after each filament to avoid cross-contamination of the various indicator powders. Once a new filament was completely extruded, the twin-screw rotation speed was increased to 100 rpm and the instrument was flushed with purge compound at 180 °C. After flushing, the heat was turned off and the water chiller was activated to quickly cool the instrument while continuing to add the purge compound. When the temperature dropped low enough so that the purge compound began to solidify within the chamber and the maximum torque (14 Nm) was reached by the screws, the machine was powered off and the chamber was opened so that the partially cured purge compound could be pulled from the chamber and mixing screws, removing any remaining residue. 

### 2.3. Indicator Testing

**Figure 3:**
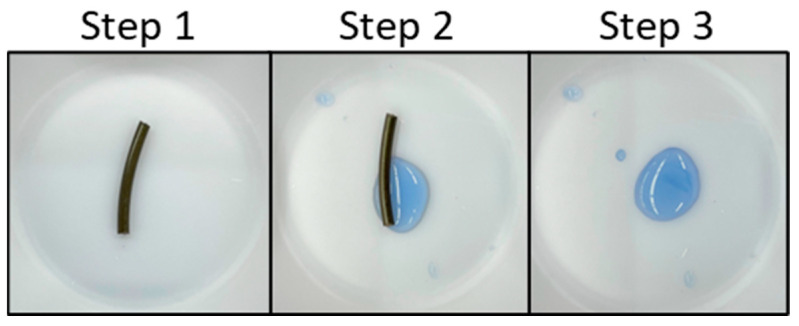
Acid/base indication testing procedure example with PLA-PEG-TB. Step 1: filament sample is placed in a plastic weigh boat; step 2: 500 µL of pH 13 solution is added to the filament sample; step 3: after 15 min, the filament is removed and the color of the solution is recorded.

To test the pH-indicating efficacy of the filaments, solutions were made with pH values of 0, 6, 7, 8, and 13, allowing the evaluation of the effectiveness of the indicting filaments when exposed to strong acids, strong bases, and neutral solutions. These pH values also reflect the most beneficial pH ranges for each of the indicators used (pH 6–8 for bromothymol blue; pH > 8 for phenolphthalein; and pH 0–14 for thymol blue). A total amount of 500 µL of drops of each solution were placed on the surface of samples of each indicating filament, and the resulting color change was recorded after a maximum of 15 min (Figure 3). Additionally, the pH indicator powders were tested with the same solutions to determine their efficacies before and after heat treatment at the highest extrusion temperature used for the filaments (180 °C).

### 2.4. Characterization

#### 2.4.1. Physical Characterization

Filaments were physically characterized via lightbox photography (to ensure even lighting for color comparisons) and optical microscopy. A confocal microscope (Keyence VHX-7000; Keyence Corporation, Osaka, Japan) was used to visually inspect the filaments before and after acid/base exposure and degradation. Additionally, hydrophobicity was analyzed by melting representative samples of each filament so that a flat surface was available for contact angle measurements. After a flat sample was created, filament samples were positioned approximately 4 mm from the syringe of a Drop Shape Analyzer (DSA30E, Krüss Scientific, Hamburg, Germany), and 10.5 µL droplets of DI water were dispensed onto the material. Contact angles were then determined by the instrument. 

#### 2.4.2. Chemical Characterization

Chemical characterization of the filaments was accomplished with Fourier transform infrared spectroscopy (FTIR), NMR, and laser-induced breakdown spectroscopy (LIBS). Absorption spectra were obtained in attenuated total reflectance (ATR) mode using a Nicolet iS50 FTIR instrument (Thermo Fisher Scientific, Waltham, MA, USA) equipped with a diamond crystal reference. Scans measured the absorbance from 525 to 4000 cm^−1^ at a resolution of 4 cm^−1^, and spectra reported here are background-subtracted and averaged from 32 scans per sample. For NMR, samples were dissolved in deuterated chloroform over 48 h, and the resulting solutions were extracted with filtered syringes and placed in NMR tubes. Extracts were analyzed via liquid-state ^1^H NMR experiments using a Bruker Avance NMR spectrometer (Bruker Corporation, Billerica, MA, USA) operating at 500.13 MHz, and residual protons in the solvent were used as a proton reference. Elemental makeup and distribution of the filaments were determined via a LIBS-based elemental analyzer (Keyence EA-300 VHX Series; Keyence Corporation, Osaka, Japan) attached to a Keyence VHX-7000 microscope. The spot size for LIBS analyses was 10 µm and element concentrations were averaged from three analyses per spot. 

#### 2.4.3. Thermal Characterization

TGA was performed on representative samples of each filament using a TA Discovery Series TGA550 (TA Instruments, New Castle, DE, USA). For these experiments, samples weighing 5–10 mg were cut from filaments and heated from 25 to 750 °C at a rate of 10 °C/min. During analysis, ultra-high purity nitrogen gas was flowed across each sample at 40 mL/min, and the masses of the samples were tracked by the instrument. The onset of thermal degradation was defined as the point at which the sample lost 5% of its starting mass (*T_d5%_*); the decomposition temperature (*T_dMax_*) was defined as the temperature at which the derivative TGA (DTGA) curve was at a local maximum; and the final mass (*m_f_*) was defined as the residual mass of the sample at the end of the experiment. All of these values (*T_d5%_*, *T_dMax_*, and *m_f_*) were determined by the Trios software (TA Instruments) associated with the TGA instrument. 

DSC analyses were conducted on ~5 mg samples of each filament using a TA Instruments DSC Q20 series (TA Instruments, New Castle, DE, USA). Samples were heated from 40 °C to 400 °C at a rate of 10 °C/min under a 5 mL/min flow of ultra-high purity nitrogen. The glass transition temperature (*T_g_*) and the melting point (*T_m_*) were determined by the Trios software, and the enthalpy of melting (Δ*H_m_*) was calculated from the area between the melting curve and the scan baseline. The percent crystallinity (*X_C_*) of the PLA in each filament was determined using Equation (1):

(1) $$X_{C}=\frac{\Delta H_{m}}{w\Delta H_{m}^{\prime }}\times 100,$$

where *w* is the weight percent of PLA in the filament and Δ*H’_m_* is the theoretical enthalpy of melting for PLA (93.6 J/g). 

#### 2.4.4. Mechanical Characterization

Mechanical characterization was performed using tensile testing with an INSTRON 3343 Low-Force Testing System with BlueHill Universal software (Version 4.08; INSTRON, Norwood, MA, USA). Samples of each filament were cut to 5 cm lengths and held in place by Screw Side Action Tensile Grips (INSTRON, Norwood, MA, USA) attached to the testing system. The gripped samples were then extended at a rate of 8.333 × 10^−3^ mm/min until breakage occurred. At least three tensile tests were completed for each filament. The resulting stress versus strain curves were averaged, and the stress at break, maximum stress, maximum strain, and Young’s modulus were determined by the associated BlueHill Universal software. 

### 2.5. Degradation Experiments

To assess the effects of the added fillers on the degradation of PLA filaments, samples of each filament were aged in two environments at 30 °C for 42 days following procedures similar to those used in [35]. The first environment was a soil slurry made up of 4.5 cc natural grassland soil (collected near Española, NM), 20 mL deionized (DI) water, and 1 cc gelatin (which has reportedly been used to selectively enhance the growth of PLA-degrading microorganisms [45,46]). The soil slurry and filament samples were placed in small glass jars capped with loose-fitting screw-top lids (to allow air permeation) and were agitated in a shaking incubator rotating at 100 rpm. Slurry samples were topped with DI water when evaporation was noticed, and pH was monitored with pH strips throughout the experiment. The second aging environment consisted of samples being buried in the same natural soil, placed in a static incubator heated to 30 °C, watered to saturation every 2–3 days, and gently mixed weekly. Following aging, samples were removed from the slurry and soil environments and gently washed by submerging in DI water before analyzing samples via microscopy, FTIR, and DSC. After initial microscope images were taken, samples were further washed by vortexing in DI water to better visualize the extent of pitting on each filament. 

## 3. Results and Discussion

### 3.1. Physical Characterization

**Figure 4:**
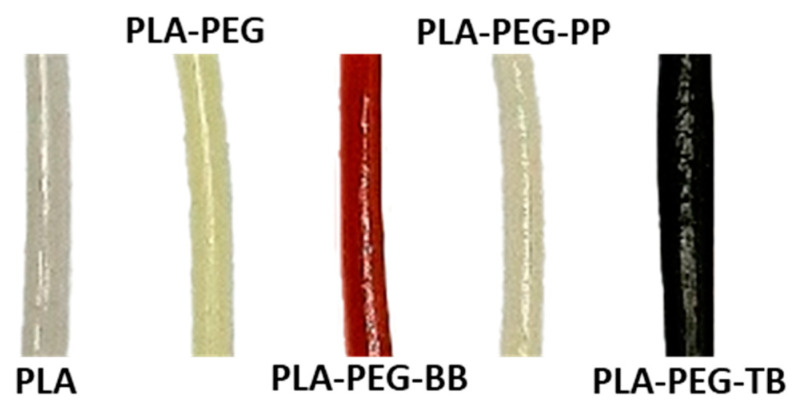
Filaments created in this study.

The colors of the filaments created here were the result of the fillers added as well as the temperature of extrusion. Overall, the indicators appeared to be evenly distributed throughout the indicator filaments. The PLA filament was colorless and translucent with very little difference between the filament and the original PLA pellets. The PLA-PEG filament was also translucent, but it was slightly yellowed compared to the PLA and PEG raw materials (Figure 4). This yellowing was likely due to impurities within the PEG prior to filament fabrication. The PLA-PEG-BB filament was the most translucent of the PLA-PEG-indicator filaments, suggesting lower crystallinity compared to the PLA-PEG-PP and PLA-PEG-TB filaments, which was confirmed by DSC analyses discussed later in this paper. Compared to the bromothymol blue indicator powder, the PLA-PEG-BB filament was darker and redder (Figure 1 and Figure 4); this change in color was attributed to the elevated temperature used to create the filament (180 °C), which is near the melting temperature of the indicator (204 °C), rather than an indication of pH during filament compounding and extrusion. To test this hypothesis, raw bromothymol blue powder was heated in an oven for two hours at 180 °C. After removal from the oven, the powder was partially melted and had become darker and redder, similar to the coloration of the PLA-PEG-BB filament. The PLA-PEG-PP filament was slightly opaque, suggesting higher crystallinity than the PLA, PLA-PEG, and PLA-PEG-BB filaments—which was also later confirmed by DSC analyses—and its color was very close to that of the phenolphthalein indicator powder (Figure 1 and Figure 4). Finally, the PLA-PEG-TB filament was also opaque, again suggesting higher crystallinity than the translucent filaments which was confirmed via DSC analyses. This filament was a very dark, earthy green, which is slightly different from the lighter green-yellow-brown color of the raw thymol blue (Figure 1 and Figure 4). The role of the elevated temperature during extrusion on the coloration of the PLA-PEG-TB filament was investigated by heating the indicator powder to 180 °C for two hours. This treatment did not result in melting or a color change of the indicator, suggesting that temperature was not the cause of the filament coloration. Instead, it may be the result of a slightly basic environment created by the mixture and extrusion of the PLA-PEG-TB filament, causing the powder to undergo a partial pH-based reaction leading to parts of the indicator turning dark blue and leaving the rest of the indicator the original, lighter green-yellow color. When the two colors mix, they produce a filament that is dark green. 

Hydrophobicity and hydrophilicity describe the wettability of a material’s surface, with hydrophobic surfaces repelling water and hydrophilic surfaces attracting or absorbing water. These attributes are measured via contact angle analysis, where materials are traditionally classified as hydrophilic when they have static contact angles <90° and hydrophobic when the static angles are >90° [47]. Results of static contact angle measurements for the filaments in this study show that all filaments lie in the transition range between the traditional definitions for hydrophobicity and hydrophilicity (~85°–91°; Table 4), suggesting that they are neither significantly hydrophobic nor hydrophilic. Relative to the filaments within this study, PLA had the highest degree of hydrophobicity with a contact angle of 90.8° and PLA-PEG-PP had the highest degree of hydrophilicity with a contact angle of 84.8°. While PLA is generally highly hydrophobic, the addition of PEG often increases the hydrophilicity of the PLA composite [48], which appears to be the case with the PLA-PEG and PLA-PEG-indicator composite filaments created here.

### 3.2. pH Indication of Filaments

**Figure 5:**
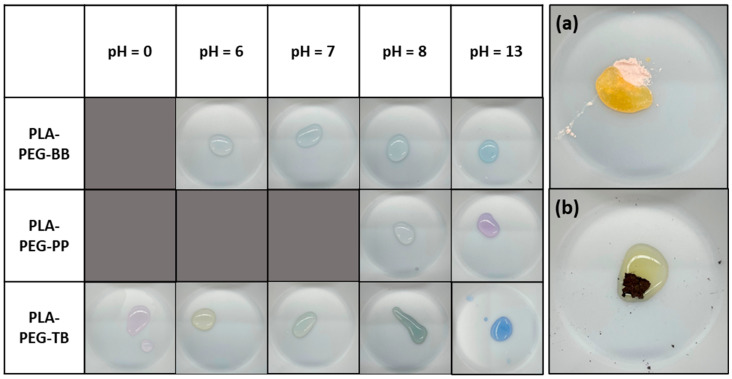
Results of indicating filaments and bromothymol blue pH tests. Side panel: (**a**) unmodified and (**b**) heat-treated bromothymol blue indicator powder exposed to pH 6 solution.

The pH exposure tests resulted in no obvious change in the color of the filaments themselves, but they did show successful surface sensing properties for nearly every filament (Figure 5 and Table 5). When acidic, neutral, and basic solutions were introduced to the indicating filaments, the solution color changed as expected for each pH, with the exception of PLA-PEG-BB at pH 6 (Figure 5 and Table 5). Solutions in contact with PLA-PEG-TB filament exhibited the widest range of color changes, corresponding to the wide pH range of the thymol blue indicator (Table 1 and Figure 5). At pH 0, PLA-PEG-TB turned the solution a light red-purple; at pH 6, the solution became yellow; at pH 7, the solution was yellow-green; at pH 8, the solution was a dark blue-green; and at pH 13, the solution was a royal blue color (Figure 5). For PLA-PEG-PP, the pH 8 solution did not result in a color change; however, this was not unexpected as phenolphthalein does not usually indicate until a solution is >pH 8 (Table 1 and Figure 5). At pH 13, PLA-PEG-PP turned the solution a bright fuchsia, corresponding to the expected color change of phenolphthalein in highly alkaline conditions. Finally, PLA-PEG-BB had the least noticeable reaction to solutions of different pH values. At pH 7, PLA-PEG-BB turned the solution a light green-blue; at pH 8, the solution turned light blue; and at pH 8, the solution became a medium blue, corresponding to the color changes expected for bromothymol blue exposed to the same pH levels (Figure 5 and Table 1). At pH 6, PLA-PEG-BB did not change the color of the solution (Figure 5). According to the indicating range of bromothymol blue, contact with a pH 6 solution should turn the mixture yellow (Table 1). This failure to indicate is likely the result of the bromothymol blue partially melting during filament compounding and extrusion, as the bromothymol blue powder heated to 180 °C exhibited a considerably muted color change when exposed to pH 6 solution relative to the color change seen in unmodified bromothymol blue powder at the same pH (Figure 5a,b).

### 3.3. Chemical Characterization

**Figure 6:**
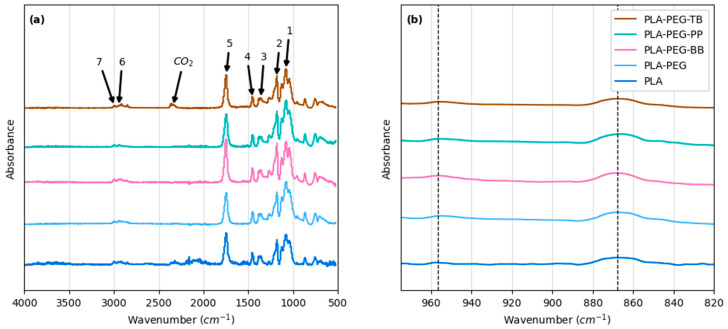
FTIR spectra of filaments created in this study at: (**a**) 500–4000 wavenumbers and (**b**) 820–970 wavenumbers. Labeled arrows correspond to peaks identified in Table 6. Dashed lines denote peaks at 956.5 cm^−1^ and 868 cm^−1^.

The chemical structure of the PLA and PLA composite filaments was investigated through FTIR. Figure 6 shows the absorbance spectra for all filaments, with the only definitive peaks attributed to functional groups within PLA or air (Figure 6a and Table 6). Though background spectra were taken before and subtracted from sample spectra, the PLA-PEG-TB sample spectra recorded a peak at 2340 cm^−1^, associated with CO_2_ in the environment (Figure 6). Peaks indicative of PEG or the indicator powders were not seen in the filament spectra. Though this is most likely because the concentrations of PEG and the indicator powders were very low (10 wt% and 5 wt%, respectively) compared to that of PLA (85–90 wt%), it also suggests that the PLA, PEG, and indicators did not form new bonds resulting from a chemical reaction among the components. In their work, Gorrasi et al. [49] reported that the differences in FTIR peak heights at 862–872 and 920 cm^−1^ represented higher and lower relative crystallinities in PLA when spectra were scaled so that peaks at 956.5 were the same height. This phenomenon was investigated here, and the results are shown in Figure 6b, where the dashed lines signify peaks at 956.5 cm^−1^ (the scaling peak) and 868 cm^−1^ (a peak assigned to flexural C-H bond vibration within PLA crystals). The FTIR scans of the all filaments revealed no crystallization peak at 920 cm^−1^ and a similarly sized crystallization peak at 868 cm^−1^, suggesting that PLA crystallinity was comparable for all filaments (Figure 6b). While FTIR may serve as a reference for estimating relative degrees of PLA crystallinity, the exact degree of crystallinity for the filaments is more precisely determined via DSC analysis, which is presented in Section 3.4. Though filament samples were also analyzed via ^1^H NMR, these analyses did not provide any additional chemical information compared to the FTIR analyses (Figure S1).

**Figure 7:**
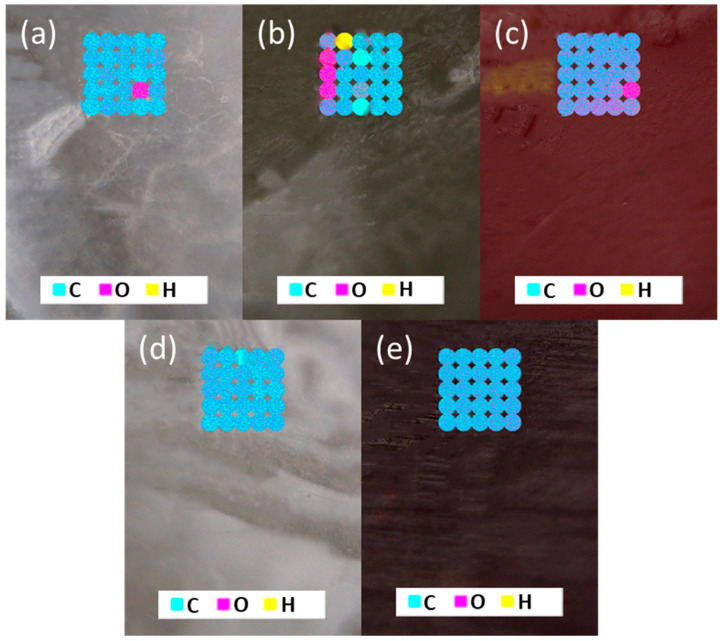
Microscope images and elemental maps of: (**a**) PLA, (**b**) PLA-PEG, (**c**) PLA-PEG-BB, (**d**) PLA-PEG-PP, and (**e**) PLA-PEG-TB filaments. Maps on each sample show 25 LIBS analyses in 5 × 5 grids.

LIBS analyses of the filaments showed no identifying elements associated with the indicator powders (i.e., S for thymol blue and bromothymol blue or Br for bromothymol blue; Figure 7). This is likely due to the low concentration (5 wt%) of the indicators compared to the PLA (85 wt%) and PEG (10 wt%). LIBS analyses found evidence for the presence of water in the PLA, PLA-PEG, and PLA-PEG-BB filaments (represented by the pink spots in Figure 7) but not in the PLA-PEG-PP and PLA-PEG-TB filaments. These results suggest that the former filaments were more susceptible to water intrusion than the latter filaments, and thus may be more vulnerable to hydrolytic degradation (the primary mechanism for PLA degradation in the environment). These results are contrary to the contact angle results reported in Table 3, which indicated that the PLA filament was the most hydrophobic and the PLA-PEG-PP filament was the most hydrophilic. This may indicate that any water adsorbed by the original filaments was lost upon melting for the contact angle measurement. 

### 3.4. Thermal Characterization

**Figure 8:**
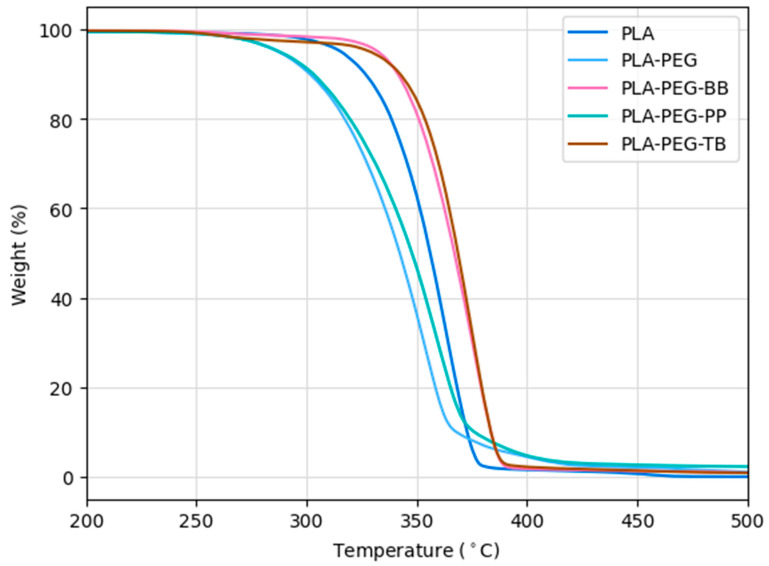
TGA results for filaments created in this study.

Consistent with previous studies [30,35], TGA confirmed that the addition of PEG lowered the thermal stability of PLA (Figure 8 and Table 7). Interestingly, the addition of pH indicators along with PEG had varied effects on PLA’s thermal stability. PLA-PEG-BB and PLA-PEG-TB filaments both had higher degradation temperatures (*T_d95%_*) than the PLA-only filament, while the PLA-PEG-PP filament had a lower degradation temperature, similar to that of PLA-PEG (Figure 8 and Table 7). 

**Figure 9:**
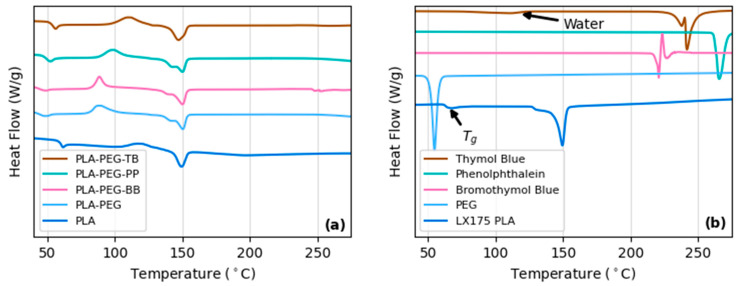
DSC results for (**a**) filaments created and (**b**) raw materials used in this study. Arrows in plot (**b**) indicate the *T_g_* of LX175 PLA and water loss in thymol blue.

Additional thermal transitions, i.e., *T_g_* and *T_m_*, and crystallinity were determined via DSC. While the melting temperatures of all filaments were similar (~150 °C), the *Tg* and the crystallinity changed with the addition of PEG and indicator powders (Figure 9a and Table 6). The addition of PEG lowered the *T_g_* of the PLA filament by ~10 °C, consistent with results seen in other studies [30,31,35,40,50]. PLA-PEG-BB and PLA-PEG-PP similarly lowered *T_g_*, suggesting that the glass transition temperature in these filaments was primarily driven by the addition of PEG rather than the indicator powders. The PLA-PEG-TB filament had a *T_g_* in the middle of those of the PLA-only filament and the other filaments (Table 7), suggesting that thymol blue may increase the *T_g_* of PLA-PEG composites. Of the raw materials used, only the LX-175 PLA pellets had a measurable glass transition temperature of 65 °C (Figure 9b). The step at 117 °C in the thymol blue DSC plot is attributed to water loss (Figure 9b). 

Exothermic reactions indicated by upward peaks in Figure 9a suggest all filaments had increased crystallinity compared to the PLA-only filament. This is confirmed by the Xc values determined by the enthalpy of melting obtained from the DSC measurements (Table 8). For the PLA-PEG, PLA-PEG-BB, and PLA-PEG-TB filaments, the increased crystallinity can likely be attributed to the PEG [30,31,35,40,50,51,52,53,54,55]. However, the crystallinity of the PLA-PEG-PP filament is ~10% higher than that of PLA-PEG and double that of PLA alone (Table 8). For this filament, the higher crystallinity may be a result of phenolphthalein acting as a nucleation center for crystal formation [35,36,37]. Double melting peaks seen in the PLA-PEG, PLA-PEG-BB, and PLA-PEG-PP filaments correspond to the formation of different crystal structures within the PLA, with the higher temperature peak representing the α-form (pseudo-orthorhombic, pseudo-hexagonal, or orthorhombic) and the lower temperature representing the β-form (orthorhombic or trigonal) [36,37,51,52]. These changes in crystallinity are likely a major factor leading to the differences seen in the biodegradation of the PLA-PEG and PLA-PEG-indicator filaments compared to PLA-only filaments, as discussed below. 

### 3.5. Mechanical Characterization

**Figure 10:**
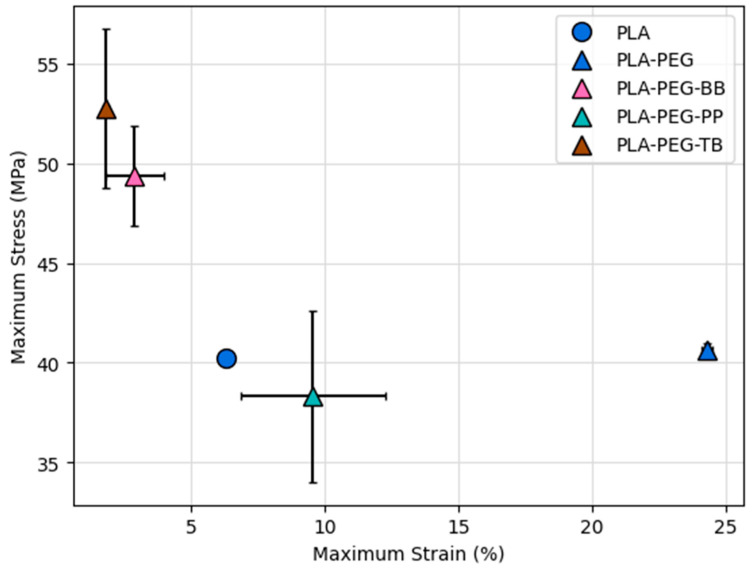
Maximum stress versus strain for filaments created in this study.

The addition of PEG usually leads to a more ductile PLA filament with lower tensile strength, while the addition of fillers such as clays and nanocomposites typically results in a more brittle material with higher tensile strength [31,40,53,54,55,56,57,58]. For the filaments in this study, the addition of 10 wt% PEG did not change the tensile strength compared to PLA, but it did increase the filament’s ductility (Figure 10 and Table 8). PLA-PEG-indicator filaments were universally less ductile than PLA-PEG, but the PLA-PEG-BB and PLA-PEG-TB filaments were also more brittle than PLA, while PLA-PEG-PP was less brittle compared to the PLA-only filament. Similarly, the addition of bromothymol blue and thymol blue to PLA-PEG filaments led to an increase in tensile strength compared to PLA and PLA-PEG filaments, while the addition of phenolphthalein had no impact on tensile strength (Figure 10 and Table 9). Tensile strength and ductility are expected to correlate with crystallinity, with higher crystallinity leading to a greater tensile strength and lower ductility. However, this was not the case with many of the filaments created here. As expected, due to the higher degrees of crystallinity compared to the PLA-only filament (Table 8), the tensile strength and brittleness of PLA-PEG-BB and PLA-PEG-TB filaments increased relative to PLA (Figure 10 and Table 9). In contrast, while PLA-PEG and PLA-PEG-PP filaments also have higher crystallinity compared to PLA (Table 8), they are more ductile than the PLA-only filament (Figure 10 and Table 9). 

### 3.6. Degradation Results

**Figure 11:**
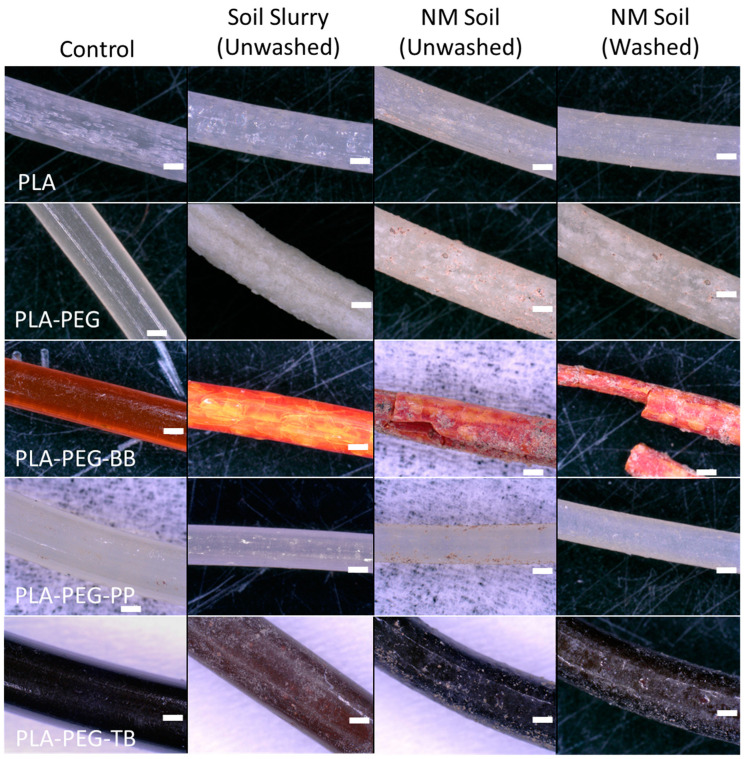
Microscope images of unaltered and biodegraded filaments created in this study. White scale bars represent 500 µm.

Microscopy of filament samples following aging via burial in natural soil or submersion in a soil slurry revealed noticeable differences in the degrees of degradation based on filament composition and aging methods (Figure 11). The PLA-only filament had some evidence of pitting and microbial growth when aged in the soil slurry. When buried and aged in NM soils, the PLA filament had wear that was more indicative of physical damage from contact with soil grains (likely a result of the weekly gentle mixing of filaments and soils) than microbial degradation, which is expected, as microbial involvement in PLA degradation is usually very minimal compared to abiotic hydrolysis and occurs much later in the biodegradation process [3,6]. Additionally, very few grains remained on the sample following removal from the soil. These results will serve as a comparison point for the remaining filament samples.

The surface of the PLA-PEG filament aged in the soil slurry showed “flaking” and was whiter than the control sample, though it had less evidence of surface microbial growth compared to the slurry-aged PLA-only filament (Figure 11). The lack of microbial colonization on this sample indicates—as expected for PLA degraded under non-composting conditions—that the degradation was solely the result of abiotic hydrolysis reactions altering the surface of the sample, degrading the amorphous regions and leaving the surface more crystalline, resulting in the flaky appearance. In contrast to the PLA-PEG filament aged in the soil slurry, the sample aged in NM soils exhibited more evidence of microbial interaction with the filament in the form of pitting and slight biofilm formation on the surface. Similar to the PLA-PEG filament aged in the soil slurry, the soil-aged PLA-PEG filament also became whiter and less translucent compared to the control sample, possibly due to a loss of amorphous material and a relative increase in crystallinity. 

The slurry-aged PLA-PEG-BB showed little to no evidence of microbial colonization, though there was a clear change in the filament’s appearance from a translucent orange to an opaque redder orange with yellow mottling and small cracks throughout the sample (Figure 11). Again, the increase in opacity likely points to an increase in crystallinity, though the mottling may be the result of the bromothymol blue indicating a local drop in pH due to PLA hydrolysis [6,49]. Similar yellow mottling and increased opacity was seen in the NM soil-incubated PLA-PEG-BB filament. This sample was visibly the most degraded filament amongst all types and conditions tested, with extensive cracking, dehydration, and brittleness, likely resulting from the removal of amorphous material through PLA hydrolysis. There was a clearly evident biofilm covering the filament, suggesting that the destruction of the filament surface attracted microorganisms within the soil. 

Unlike the other PLA-PEG filaments, PLA-PEG-PP samples did not exhibit increased opacity upon aging in soils or soil slurries, suggesting the crystallinity of this filament did not increase (Figure 11). The PLA-PEG-PP filament was less visibly degraded than the PLA-only filament when aged in the soil slurry and showed no evidence of microbial colonization. However, when aged in NM soil, the PLA-PEG-PP filament exhibited slight microbial colonization, though to a lesser extent than the other PLA-PEG filaments. 

The PLA-PEG-TB filaments showed little evidence of microbial attachment after either aging in the soil slurry or soil, though there were changes in the surface appearance of the filament similar to that seen in the PLA-PEG and PLA-PEG-BB filaments (Figure 11). The fine white coating that appeared on both PLA-PEG-TB samples was likely the result of increased crystallinity due to hydrolysis, while the lighter, redder color seen in the slurry-degraded sample may be the result of the thymol blue indicating a local drop in pH, similar to the yellow mottling seen in the degraded PLA-PEG-BB samples. 

**Figure 12:**
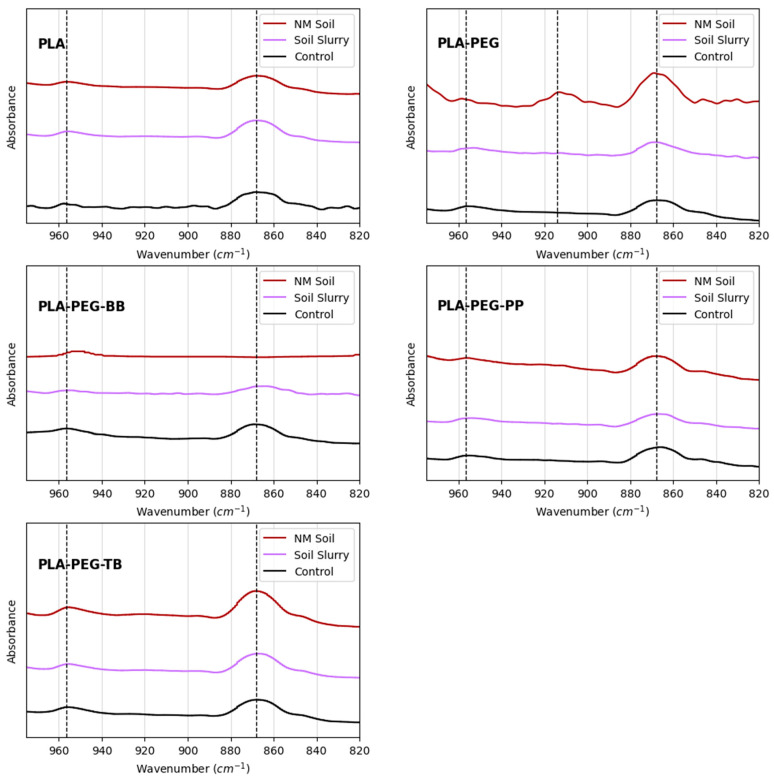
FTIR results for unaltered and biodegraded filaments created in this study. Dashed lines denote peaks at 956.5 cm^−1^, 914 cm^−1^, and 868 cm^−1^.

At first glance, few difference were noted between the FTIR traces for aged (soil slurry or soil) filaments compared to those of the control, un-aged samples. However, upon closer inspection of the lower wavenumber region, differences in peaks denoting crystallization were detected (Figure 12). The crystallization peak at 868 cm^−1^ increased following incubation in NM soil but not in soil slurries for PLA-PEG, PLA-PEG-PP, and PLA-PEG-TB filaments compared to the un-aged controls. Additionally, a peak at 914 cm^−1^ appeared in the PLA-PEG sample after aging in NM soil, also signifying an increase in PLA crystallinity [49]. For the PLA-PEG-BB filament, the 868 cm^−1^ crystallization peak decreased upon aging in the soil slurry and disappeared entirely after incubation in the NM soil. Unlike the modified PLA filaments, the 868 cm^−1^ crystallization peak for the PLA-only filament remained similar before and after aging. 

**Figure 13:**
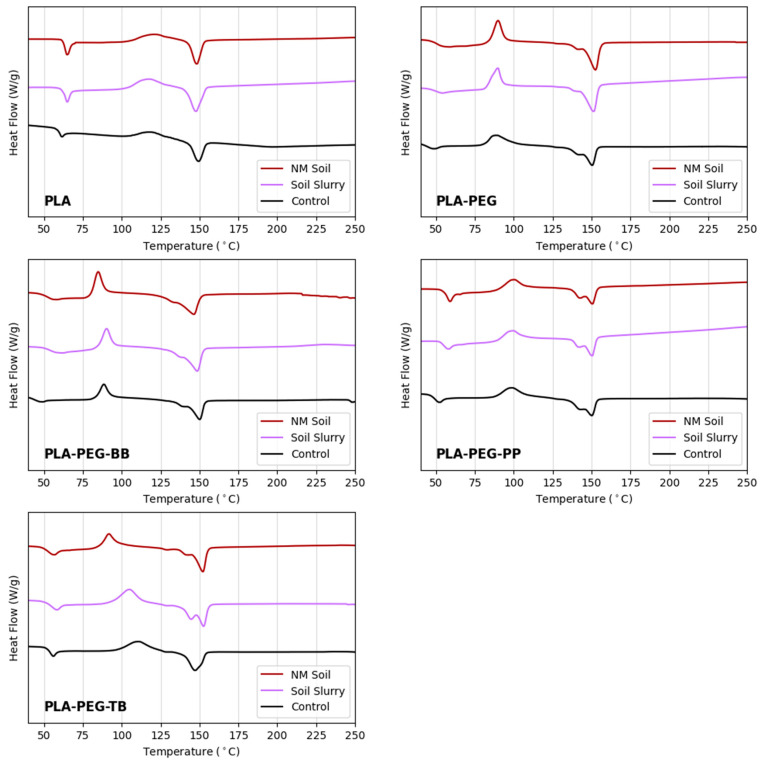
DSC results for slurry- and soil-aged PLA filaments and unaged controls.

While the physical examination and FTIR results pointed toward changes in crystallinity following degradation of the modified PLA filaments, DSC analyses revealed the actual changes in crystallinity and thermal transitions resulting from incubation in soil slurry and NM soil. Overall, crystallinity increased in aged samples compared to control samples, with the largest crystallinity increases seen in the PLA-PEG-BB filaments, suggesting these filaments experienced the most hydrolysis during degradation (Figure 13 and Table 10). Conversely, PLA-PEG-PP filaments experienced a decrease in crystallinity following degradation. This is consistent with the unchanging transparency of the control and degraded samples seen in the microscope images in Figure 11 and points to a loss of crystalline material rather than amorphous material. For the PLA-PEG-TB filament, there was a small increase in crystallinity, resulting from degradation in the NM soil and a much larger increase in crystallinity resulting from degradation in the soil slurry (Table 10), suggesting that hydrolysis proceeded further in the slurry than in the NM soil. Additionally, the presence of double melting peaks, indicative of the presence of multiple PLA crystal forms [36,37,51,52], in both the slurry- and soil-aged PLA-PEG-TB samples suggest that the degree of hydrolysis and subsequent increase in crystallinity may be related to the heterogeneity of crystal forms in PLA-PEG-TB composites. The increase in *T_g_* for the slurry-aged PLA-PEG and slurry- and soil-aged PLA-PEG-BB was consistent with an increased proportion of crystalline PLA in these filaments following degradation (Figure 13 and Table 10). 

## 4. Conclusions

In this study, PLA was melt-blended with PEG, bromothymol blue, phenolphthalein, and thymol blue to produce pH-indicating functional filaments for additive manufacturing. Overall, the filaments were able to indicate exposure to solutions of various pH values, with the exception of PLA-PEG-BB, which functioned only as a base indicator due to heat alteration of bromothymol blue during filament extrusion. Chemical, thermal, and mechanical analyses of the new filaments revealed that the addition of indicator powders increased filament crystallinity by 14–22% compared to PLA-only filaments, while changes in thermal stability and mechanical characteristics depended upon the indicator used; PLA-PEG-BB and PLA-PEG-TB filaments were more thermally stable, had higher tensile strength, and were less ductile than PLA-only filaments, while PLA-PEG-PP filaments were less thermally stable, had lower tensile strength, and were more ductile. Finally, the effects and rates of degradation in soils and soil slurries were also influenced by the indicator composition, with the physical deterioration and changes in filament crystallinity suggesting that the PLA-PEG-BB filament degrades faster than PLA-only filaments, while the PLA-PEG-PP and PLA-PEG-TB filaments saw decreases in degradation rates.

## Figures and Tables

**Table 1:** Physical properties of indicator powders used in this study.

Indicator	Melting Point (°C)	Solution pH	Solution Color Change
Bromothymol Blue	204	6–6.5	Yellow
		6.5–7	Green
		>7	Blue
Phenolphthalein	260–263	>8	Pink/Fuchsia
Thymol Blue	221	<2.8	Red/Purple
		2.8–9.0	Yellow
		>9	Blue

**Table 2:** Names and compositions for filaments created in this study.

Name	Weight % PLA	Weight % PEG	Weight % BB	Weight % PP	Weight % TB
PLA	100	0	0	0	0
PLA-PEG	90	10	0	0	0
PLA-PEG-BB	85	10	5	0	0
PLA-PEG-PP	85	10	0	5	0
PLA-PEG-TB	85	10	0	0	5

**Table 3:** Extrusion temperatures used for filaments in this study.

Filament	Extrusion Temperature (°C)
PLA	180
PLA-PEG	170
PLA-PEG-BB	160
PLA-PEG-PP	170
PLA-PEG-TB	180

**Table 4:** Results of contact angle measurements. The reported error reflects the published accuracy of the instrument (±2.0°).

Filament	Contact Angle (°)
PLA	90.8 ± 2.0
PLA-PEG	88.5 ± 2.0
PLA-PEG-BB	87.7 ± 2.0
PLA-PEG-PP	84.8 ± 2.0
PLA-PEG-TB	86.8 ± 2.0

**Table 5:** Success of indicating filaments acid and base exposure tests. N = No; Y = Yes; N/A = Not Applicable.

pH	PLA-PEG-BB	PLA-PEG-PP	PLA-PEG-TB
0	N/A	N/A	Y
6	N	N/A	Y
7	Y	N/A	Y
8	Y	N (N/A)	Y
13	Y	Y	Y

**Table 6:** FTIR peaks associated with the spectra in Figure 6.

Peak Number	Wavenumber (cm^−1^)	Vibrational Mode
1	1080	C–O stretching
2	1187	C–O stretching
3	1361	Symmetric –CH_3_ bending
4	1452	Asymmetric –CH_3_ bending
5	1746	C=O stretching
6	2946	Asymmetric –CH_3_ stretching
7	2995	Symmetric –CH_3_ stretching

**Table 7:** Thermal stability properties of filaments created in this study. The reported errors reflect the published accuracy of the TGA (±1%) and DSC (±3%) instruments.

Filament	T*_d95%_* (°C)	T*_dMax_* (°C)	*m_f_*	T*_g_*(°C)	T*_m_*(°C)
PLA	315 ± 3	381 ± 4	0.065%	61 ± 2	149 ± 4
PLA-PEG	287 ± 3	367 ± 4	0.081%	50 ± 2	150 ± 5
PLA-PEG-BB	332 ± 3	392 ± 4	0.046%	50 ± 2	150 ± 5
PLA-PEG-PP	288 ± 3	398 ± 4	0.712%	52 ± 2	150 ± 5
PLA-PEG-TB	329 ± 3	393 ± 4	0.121%	57 ± 2	147 ± 4

**Table 8:** Percent crystallinity (*X_C_*) of filaments created in this study.

Filament	*X_C_*
PLA	19%
PLA-PEG	31%
PLA-PEG-BB	33%
PLA-PEG-PP	41%
PLA-PEG-TB	34%

**Table 9:** Mechanical properties of filaments created in this study. Errors reported are the standard deviation of 3–8 analyses.

Filament	Stress at Break (MPa)	Maximum Stress (MPa)	Maximum Strain	Young’s Modulus (MPa)
PLA *	18.04 ± 0.2	40.21 ± 0.4	6.32% ± 0.1%	2939 ± 29
PLA-PEG *	27.13 ± 0.3	40.61 ± 0.4	24.30% ± 0.2%	5260 ± 53
PLA-PEG-BB	46.07 ± 4.6	49.40 ± 2.5	2.89% ± 1.1%	3461 ± 421
PLA-PEG-PP	26.11 ± 7.1	38.31 ± 4.3	9.55% ± 2.7%	3206 ± 1061
PLA-PEG-TB	52.17 ± 4.0	52.76 ± 4.0	1.82% ± 0.1%	4661 ± 205

* The reported errors for these samples reflect the published accuracy of the instrument.

**Table 10:** Thermal transitions of unaged versus aged filaments. Errors reported reflect the published accuracy of the instrument (±3%). Values in parentheses represent the *X_C_* difference between the control and aged samples.

Filament	Exposure Type	T*_g_*(°C)	T*_m_*(°C)	*X_C_*
PLA	Control	61 ± 2	149 ± 4	19%
	Soil Slurry	65 ± 2	148 ± 4	* 32% (+13%)
	NM Soil	65 ± 2	148 ± 4	* 27% (+8%)
PLA-PEG	Control	50 ± 2	150 ± 5	31%
	Soil Slurry	54 ± 2	151 ± 5	* 41% (+10%)
	NM Soil	* 59± 2	152 ± 5	* 39% (+8%)
PLA-PEG-BB	Control	50 ± 2	150 ± 5	33%
	Soil Slurry	* 61 ± 2	148 ± 4	* 48% (+15%)
	NM Soil	* 58 ± 2	146 ± 4	* 49% (+16%)
PLA-PEG-PP	Control	52 ± 2	150 ± 5	41%
	Soil Slurry	* 58 ± 2	150 ± 5	* 32% (−9%)
	NM Soil	* 59 ± 2	150 ± 5	* 31% (−10%)
PLA-PEG-TB	Control	57 ± 2	147 ± 4	34%
	Soil Slurry	59 ± 2	153 ± 5	* 40% (+6%)
	NM Soil	57 ± 2	152 ± 5	37% (+3%)

* Significant change compared to control samples.

## Data Availability

The authors confirm that the data supporting the findings of this study are available within the article and associated Supplemental Information.

## Supplementary Materials

The following supporting information can be downloaded at: https://www.mdpi.com/article/10.3390/polym15020436/s1, Figure S1: ^1^H NMR results for aged and unaged filaments.
