# Supplementary figures and images for: Functional Filaments: Creating and Degrading pH-Indicating PLA Filaments for 3D Printing
Source: Polymers (Basel). 2023 Jan 13;15(2):436. doi: 10.3390/polym15020436 (PMC9866878; doi:10.3390/polym15020436)

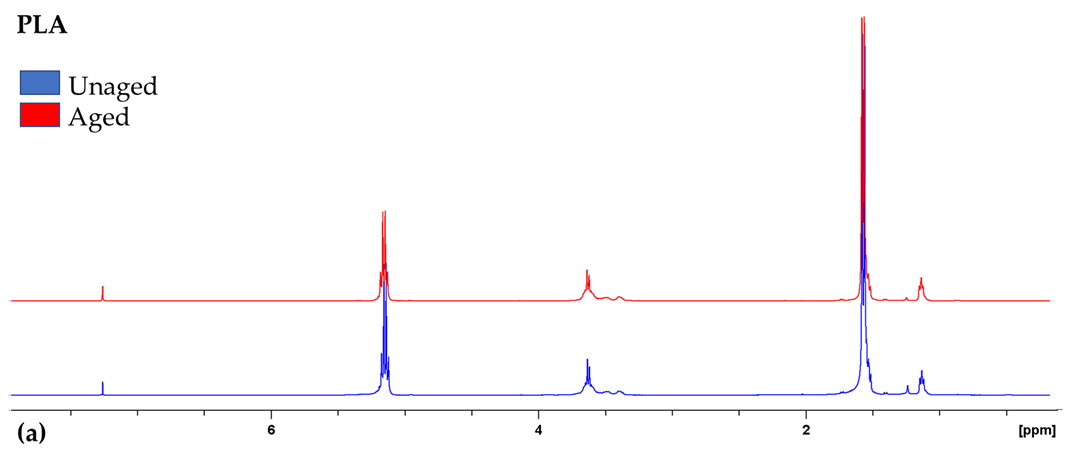

Supplement: Supplementary file 1 [file polymers-15-00436-s001.zip › Figure S1a.png]

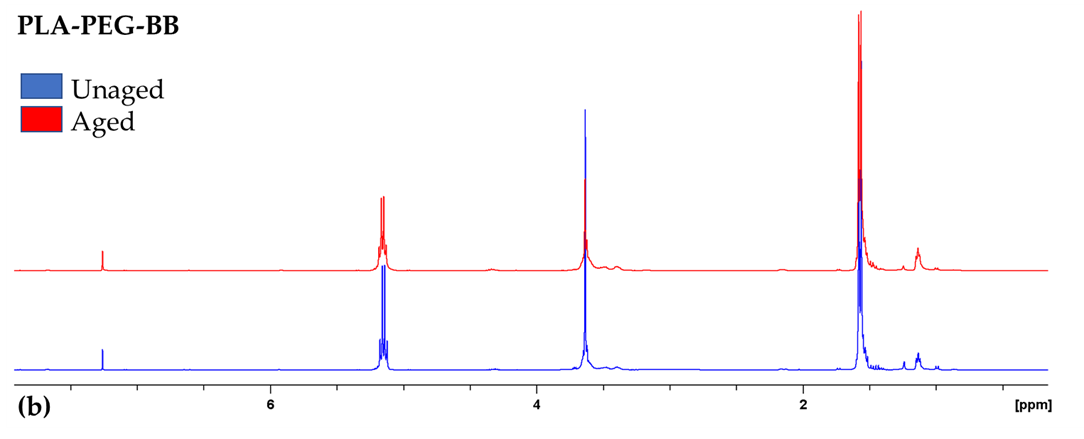

Supplement: Supplementary file 1 [file polymers-15-00436-s001.zip › Figure S1b.png]

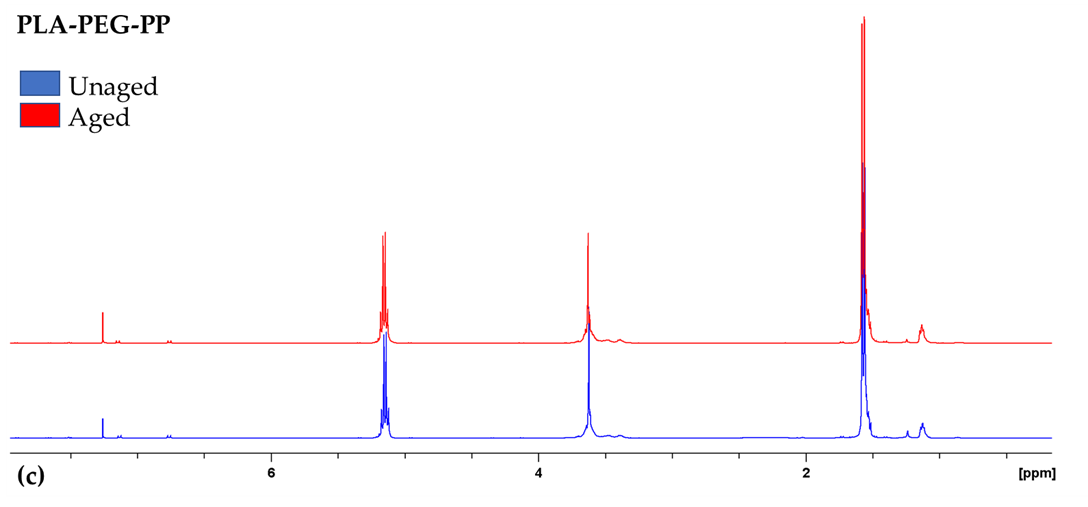

Supplement: Supplementary file 1 [file polymers-15-00436-s001.zip › Figure S1c.png]

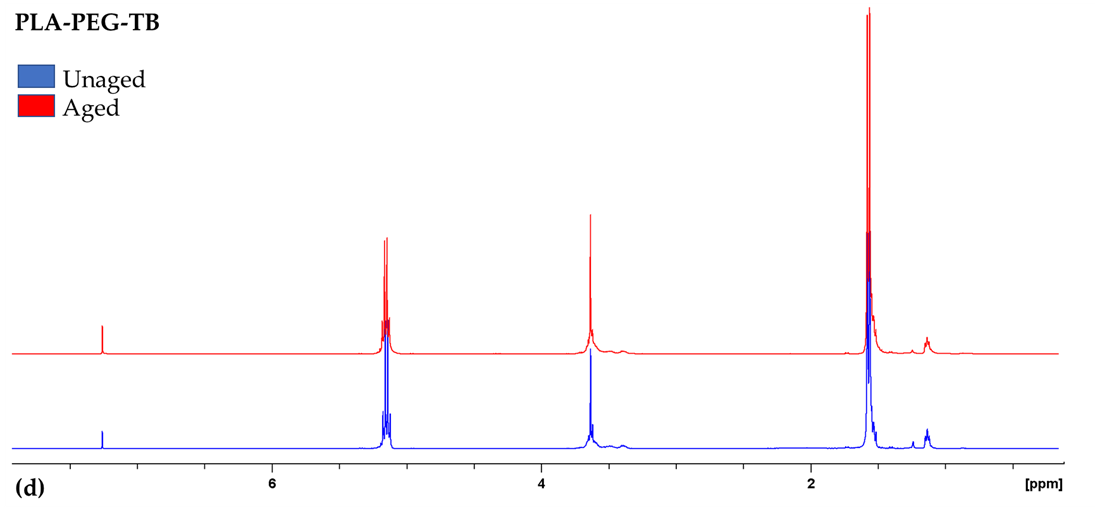

Supplement: Supplementary file 1 [file polymers-15-00436-s001.zip › Figure S1d.png]
